# Enhancing Electronic Nose Performance Based on a Novel QPSO-KELM Model

**DOI:** 10.3390/s16040520

**Published:** 2016-04-11

**Authors:** Chao Peng, Jia Yan, Shukai Duan, Lidan Wang, Pengfei Jia, Songlin Zhang

**Affiliations:** College of Electronic and Information Engineering, Southwest University, Chongqing 400715, China; pengchaocg@163.com (C.P.); duansk@swu.edu.cn (S.D.); ldwang@swu.edu.cn (L.W.); jiapengfei@swu.edu.cn (P.J.); z574066616@163.com (S.Z.)

**Keywords:** electronic nose, feature extraction, kernel extreme learning machine, quantum-behaved particle swarm optimization

## Abstract

A novel multi-class classification method for bacteria detection termed quantum-behaved particle swarm optimization-based kernel extreme learning machine (QPSO-KELM) based on an electronic nose (E-nose) technology is proposed in this paper. Time and frequency domain features are extracted from E-nose signals used for detecting four different classes of wounds (uninfected and infected with *Staphylococcu aureus*, *Escherichia coli* and *Pseudomonas aeruginosa*) in this experiment. In addition, KELM is compared with five existing classification methods: Linear discriminant analysis (LDA), quadratic discriminant analysis (QDA), extreme learning machine (ELM), k-nearest neighbor (KNN) and support vector machine (SVM). Meanwhile, three traditional optimization methods including particle swarm optimization algorithm (PSO), genetic algorithm (GA) and grid search algorithm (GS) and four kernel functions (Gaussian kernel, linear kernel, polynomial kernel and wavelet kernel) for KELM are discussed in this experiment. Finally, the QPSO-KELM model is also used to deal with another two experimental E-nose datasets in the previous experiments. The experimental results demonstrate the superiority of QPSO-KELM in various E-nose applications.

## 1. Introduction

An electronic nose (E-nose), combined with artificial intelligence algorithms, is designed for mimicking the mammalian olfactory system to recognize gases and odors. The gas sensor array in an E-nose comprises several non-specific sensors and will generate characteristic patterns when exposed to odorant materials. Patterns of known odorants can be used to construct a database and train a pattern recognition model through quite a few pattern recognition algorithms. In this way, something unknown which can be discriminated by its odor is classified well [1,2,3]. During the past decades, much work has been done to investigate the E-nose technology which has been widely used in a multitude of fields, such as food quality control [4,5,6,7], disease diagnosis [8,9,10,11], environment quality assessment [12,13] and agriculture [14,15,16].

Previous work has proved the effectiveness of detecting bacteria by investigating volatile organic compounds (VOCs) emitted from cultures and swabs taken from patients with infected wounds [17,18,19]. In the pattern recognition, firstly, training data are employed to train the classifier. Then, the performance of this classifier is assessed by using the remaining independent testing samples. The final accuracy can be computed by comparing predicted classes with their true classes. So far, various kinds of classification models have been explored in E-nose applications, which can generally be divided into two categories. One is the linear classifier, such as k-nearest neighbor (KNN) [20,21,22], linear discriminant analysis (LDA) [23,24], partial least squares regression (PLSR) [25] and Bayes classifier [26,27], which is simple and relatively easy to construct but performs poor when it deals with a host of nonlinear problems in E-nose data processing. Another is nonlinear classification models such as the multilayer perceptron (MP) [28,29], radial basis function neural network (RBFNN) [24,30] and decision tree (DT) [31]. The nonlinear classifiers can not only fully approximate the nonlinear relationship of the data, but also show exceedingly strong robustness and fault tolerance. However, they show slow convergence, require too much learning time and are liable to get trapped in local optima.

Support vector machine (SVM) is a pretty promising machine learning method that has been widely applied in classification of E-nose data, especially in some complex odor discriminations. It has better results than many classifiers, not only in qualitative and quantitative analysis of E-nose results but also in other applications [32,33,34]. Extreme learning machine (ELM), a fast learning algorithm for single hidden layer feedforward neural networks (SLFNs), first proposed by Huang *et al.* [35], randomly generates the hidden node parameters and then analytically determines the output weights instead of iterative tuning. Therefore, ELM runs fast, is easy to implement and shows superiority over other classifiers [36]. Nowadays, ELM has been widely used in a range of fields, such as sales forecasting [37], mental tasks [38], face recognition [39] and food quality tracing [40]. However, the classification performance is obviously affected by the algorithm parameters. Meanwhile, the randomly generated input weights and hidden layer biases of ELM can make the algorithm unstable [41].

Kernel Extreme Learning Machine (KELM) is constructed based on ELM combined with kernel functions in this paper considering the above limiting factors. It not only has a good deal of the advantages of ELM, but also can nonlinearly map nonlinear inseparable patterns to a separable high-dimensional feature space, which further improves the accuracy of discriminations. However, due to the existence of kernel functions, KELM is sensitive to the kernel parameters settings. Thus, the quantum-behaved particle swarm optimization (QPSO) is used to optimize the parameters of KELM and in this paper and the QPSO-KELM method is applied to improving the classification accuracy of wound infection detection. The results demonstrate that the proposed method can obtain excellent classification performance in E-nose applications.

## 2. Materials and Experiments

The datasets used in the paper were obtained by a home-made E-nose, which details can be found in our previous publication [42]. However, to make the paper self-contained, the system structure and experimental setup are briefly repeated here.

### 2.1. E-Nose System

The sensor array in the research is constructed due to the high sensitivity and quick response of the sensors to the metabolites of three different bacteria. The E-nose system consists of 15 sensors: Fourteen metal oxide gas sensors (TGS800, TGS813, TGS816, TGS822, TGS825, TGS826, TGS2600, TGS2602, TGS2620, WSP2111, MQ135, MQ138, QS-01 and SP3S-AQ2) and one electrochemical sensor (AQ sensor). A 14-bit data acquisition system (DAS) is used as interface between the sensor array and a computer. The DAS converts analog signals from sensor array into digital signals which are stored in the computer for further processing.

### 2.2. Experimental Setup

**Figure 1:**
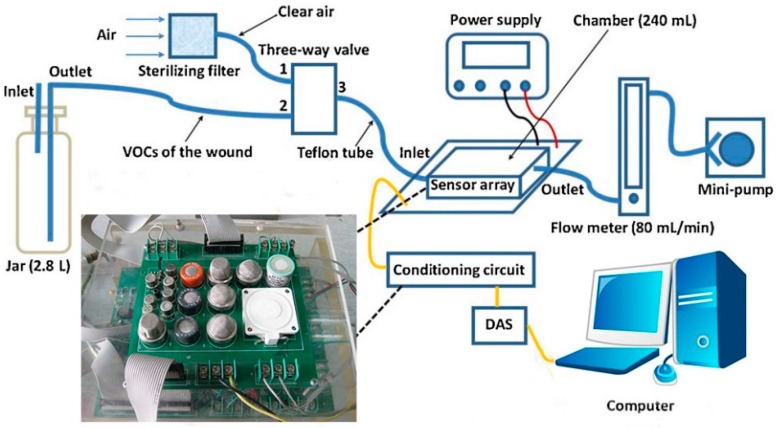
Schematic diagram of the experimental system.

Figure 1 shows the schematic diagram of the experimental system. It can be observed that the E-nose system is composed of an E-nose chamber, a data acquisition system (DAS), a pump, a rotor flowmeter, a triple valve, a filter, a glass wild-mouth bottle and a computer. The filter is used to purify the air. The pump is used to convey the VOCs and clean air over the sensor array. The rotor flowmeter is used to control the flow rate during the experiments. The three-way valve is used for switch between VOCs and clean air. The experimental setup has also been mentioned in [33]. The experimental procedure in this paper can be summarized as follows.

Each mouse was put in a big glass bottle with a rubber stopper. Two holes were made in the rubber stopper with two thin glass tubes inserted. One longer glass tube was used as an exit pipe and hung above the wound as close as possible while the shorter one was used as an intake-tube, inserted into the glass a little and was close to the bottleneck. The gases which contained the VOCs of the wound on the mouse outflowed along the longer glass tube and flowed into the sensor chamber. The air flowed into the glass along the shorter glass tube. Each test process comprises three stages: the baseline stage, the response stage and the recovery stage. In the baseline stage, the three-way valve switched on Port 1 and the clean air purified by the filter flowed through the sensor chamber for 3 min. In the response stage, the three-way valve switched on Port 2 and the gases containing the VOCs of the wound flowed through the sensor chamber for 5 min. In the recovery stage, the three-way valve switched on Port 1 again and the clean air flowed through the sensor chamber for 15 min. During the three stages of one test, the DAS always sampled the data and stored them in the computer. After one test and before the next one, for eliminating the influence of the residual odors, the sensor chamber was purged by the clean air for 5 min and in the purging process the DAS did not sample the data.

**Figure 2:**
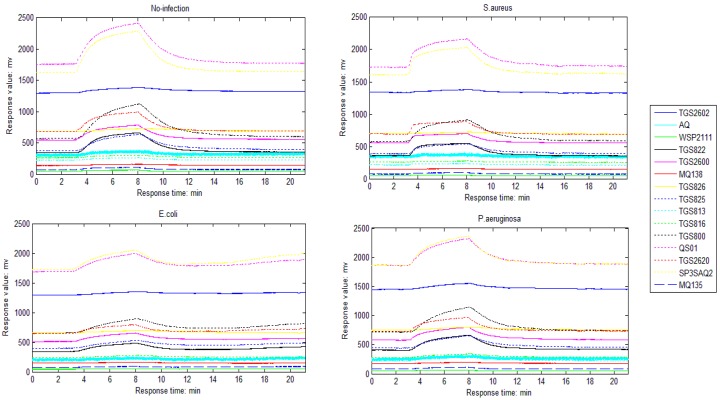
E-nose response to four wounds.

Four groups of mice were tested in the research, including one control group and three groups infected by *Staphylococcu aureus, Escherichia coli and Pseudomonas aeruginosa*, respectively. Twenty tests for each groups of mice in the same conditions were made, and finally 80 samples for all four groups of mice were collected from the above procedures. Figure 2 illustrates the sensor responses process when they are exposed to four different target wounds, where X-axis is the response time of the sensors and Y-axis is the output voltage of the sensors.

## 3. Methodology

### 3.1. KELM

ELM [36,41,43,44,45] is designed as a single hidden layer feed forward network and has been proved that its learning speed is extremely fast. It provides efficient unified solutions to generalized SLFNs, whose hidden nodes can be any piecewise nonlinear function. KELM generalizes ELM from explicit activation to implicit mapping function and produce better generalization in most applications. A brief introduction of KELM is as follows:

Suppose there are *N* training samples (***x_i_***,***t_i_***) where ***x**_i_* = [*x_i_*_1_,*x_i_*_2_,…,*x_in_*]*^T^* ∈ ***R**^n^* denotes one sample point in the *n*-dimensional space and ***t**_i_* = [*t_i_*_1_,*t_i_*_2_,…,*t_in_*]*^T^* ∈ ***R**^m^* is the sample class label. The SLFNs and activation function are defined as:

(1) $$\begin{array}{l} o_{i}=\sum_{j=1}^{L}\beta_{j}g_{j}(x_{i})=\sum_{j=1}^{L}\beta_{j}g(w_{j}\cdot x_{i}+b_{j})\\ w_{j}={\left[w_{j1},w_{j2},\cdots ,w_{jn}\right]}^{T},\beta_{j}={\left[\beta_{j1},\beta_{j2},\cdots ,\beta_{jm}\right]}^{T},i=1,\cdots ,N \end{array}$$

where ***x**_i_* is the *i*-th sample, *L* is the number of hidden nodes, ***w**_j_* and ***β**_j_* denote the input weights to the hidden layer and the output weight linking the *j*-th hidden node to the output layer respectively. Meanwhile, *b_j_* is bias of the *j*-th hidden node and ***o**_i_* is the output vector of the input sample ***x**_i_*.

Then, this SLFN can approximate those *N* samples with zero error, which means that:

(2) $$\sum_{i=1}^{L}\left\|o_{i}-t_{i}\right\|=0$$

where ***t**_i_* is the sample class label vector of the input sample ***x**_i_*. That is to say, there exist ***β**_j_*, ***w**_j_* and *b_j_* such that:

(3) $$\sum_{j=1}^{L}\beta_{j}g(w_{j}\cdot x_{i}+b_{j})=t_{i}$$

This can be written as:

(4) $${\left(\begin{array}{ccc} g(w_{1}\cdot x_{1}+b_{1}) & \cdots & g(w_{L}\cdot x_{1}+b_{L})\\ \vdots & \ddots & \vdots \\ g(w_{1}\cdot x_{N}+b_{1}) & \cdots & g(w_{L}\cdot x_{N}+b_{L}) \end{array}\right)}_{N\times L}{\left(\begin{array}{c} \beta_{1}^{T}\\ \vdots \\ \beta_{L}^{T} \end{array}\right)}_{L\times m}={\left(\begin{array}{c} t_{1}^{T}\\ \vdots \\ t_{L}^{T} \end{array}\right)}_{N\times m}$$

Then, Equation (4) can be also written as matrix form:

(5) Hβ=T

where $H=\left[\begin{array}{c} h(x_{1})\\ \vdots \\ h(x_{N}) \end{array}\right]=\left(\begin{array}{ccc} g(w_{1}\cdot x_{1}+b_{1}) & \cdots & g(w_{L}\cdot x_{1}+b_{L})\\ \vdots & \ddots & \vdots \\ g(w_{1}\cdot x_{N}+b_{1}) & \cdots & g(w_{L}\cdot x_{N}+b_{L}) \end{array}\right)$ is hidden layer output matrix.

Then, training such an SLFN is equivalent to finding a least-square solution as follows:

(6) $$\beta \prime =H^{+}T$$

where **H**^+^ is the Moore-Penrose generalized inverse of the hidden layer output matrix **H**.

Huang *et al.* suggested adding a positive value 1/*C* (*C* is regularization coefficient) to calculate the output weights as follows according to the ridge regression theory:

(7) $$\beta =H^{T}{\left(\frac{1}{C}+HH^{T}\right)}^{-1}T$$

The output function for the SLFN is:

(8) $$f(x_{i})=h(x_{i})\beta$$

where ***h***(***x**_i_*) is the output of the hidden nodes and actually maps the data from input space to the hidden layer feature space **H**.

Thus, substitute Equation (7) into Equation (8), the output function can be defined as follows:

(9) $$f(x_{i})=\left[\begin{array}{c} h(x_{i})h{\left(x_{1}\right)}^{T}\\ \vdots \\ h(x_{i})h{\left(x_{N}\right)}^{T} \end{array}\right]{\left(\frac{1}{C}+HH^{T}\right)}^{-1}T$$

We define a kernel function *k* as:

(10) $$k_{lk}=k(x_{l},x_{k})=h(x_{l})h{\left(x_{k}\right)}^{T}$$

and then a KELM can be constructed using the kernel function exclusively, without having to consider the mapping explicitly.

We express this kernel function by Equation (11) for given classes *p* and *q*:

(11) $${\left(k_{lk}\right)}_{pq}=h(x_{l}^{p})h{\left(x_{k}^{q}\right)}^{T}$$

Let **K** be a *N × N* matrix and **K** = (***K**_pq_*)_*p* = 1,2,…,*S*, *q* = 1,2,…,*S*_ where ***K**_pq_*, is a matrix composed of inner the product in the feature space:

(12) $$K={\left(K_{pq}\right)}_{\begin{array}{l} p=1,2,\ldots ,S\\ q=1,2,\ldots ,S \end{array}},K_{pq}={\left(k_{lk}\right)}_{\begin{array}{l} l=1,2,\ldots ,N_{p}\\ k=1,2,\ldots ,N_{q} \end{array}}$$

where *S* is the number of the total classes, *N_p_* and *N_q_* are the number of the samples in *p*-th and *q*-th classes respectively, ***K**_pq_* is a (*N_p_* × *N_q_*) matrix and **K** is a symmetrical matrix such that $K_{pq}^{T}=K_{pq}$.

We can define the kernel matrix **K** = **HH***^T^* from Equation (10) and the output function of KELM can be written as:

(13) $$f(x_{i})={\left(\begin{array}{c} K(x_{i},x_{1})\\ \vdots \\ K(x_{i},x_{N}) \end{array}\right)}^{T}{\left(\frac{1}{C}+K\right)}^{-1}T$$

Some common kernel functions including linear kernel function, polynomial kernel function, Gaussian kernel function, wavelet kernel function are applied. Kernel parameters of the kernel functions, together with regularization coefficient *C* in Equation (13) will be optimized by QPSO. In this way, the index of the output node with the highest output value is considered as the label of the input data [44].

### 3.2. QPSO-KELM Model

It is well known that the parameters in algorithms will affect the performances. Therefore, QPSO [46] is used to optimize the value of *C* in Equation (10) and parameters of the kernel function. The dimension of searching space is corresponding to the number of parameters of KELM with different kernel functions, and the position of each particle represents the parameter values of kernel functions. Because the best generalization performance of KELM can be optimized by QPSO, the testing accuracy can be used as the fitness function of QPSO. The specific steps of QPSO-KELM are described as follows.

*Step 1*: Normalize all the dataset extracted from the E-nose signals into the range [0,1] and the number of iterations and the population size are set as 30 and 400.
*Step 2*: Initialize the position and local optimal position of each candidate particle, as well as global best position of the swarm.
*Step 3*: Calculate each particle’s fitness value according to the fitness function. Update the local optimal positions and global best position.
*Step 4*: Update the position of each candidate particle in each iteration, which can be calculated by Equation (13).
*Step 5*: Check the termination criterion. If the maximum number of iterations is not yet reached, return to *Step 3* or else go to the *Step 6*.
*Step 6*: The best combination of parameters of the kernel function can be acquired, which result in the maximal fitness value.

**Figure 3:**
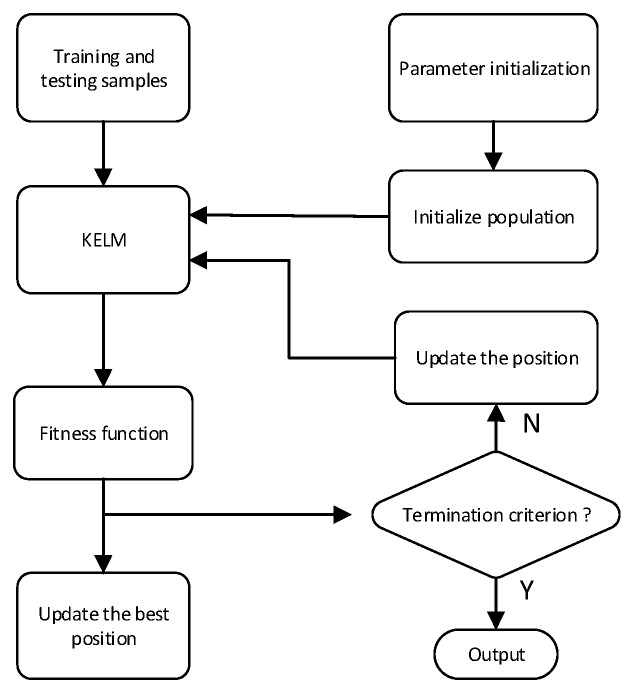
Computational procedure of QPSO for optimizing KELM.

The flowchart of this procedure is illustrated in Figure 3.

## 4. Results and Discussion

Different features which are able to effectively represent the response of sensors are extracted from the time domain and frequency domain in order to evaluate the effectiveness of the proposed model. The peak value, the integral in the response stage, coefficients of Fourier coefficients (the DC component and first order harmonic component), and approximation coefficients of db1 wavelet of sensor response curve are chosen to be on behalf of the characteristics of E-nose signals from two transform domains [47,48,49,50]. Then, leave-one-out cross validation (LOO-CV) method is employed to evaluate the performances of different methods in this experiment for making full use of the data set. Another five classification models, namely ELM, SVM, KNN, LDA and quadratic discriminant analysis (QDA), are applied for comparison with KELM. ELM is an algorithm for single-hidden layer feed forward networks training that leads to fast networking requiring low human supervision. The main idea in ELM is that the network hidden layer parameters need not to be learned, but can be randomly assigned. The only parameter is the number of hidden nodes in the hidden layer of SLFN, which is normally obtained by a trial and error method. Thus, the input weights are within (−1, 1) and the hidden layer biases are within (0, 1). 100 experiments were carried out according to the number of hidden nodes in the hidden layer from 1 to 100. Because the input weights and the hidden layer biases were chosen randomly, this experiment was repeated for 100 times. The best performance of all results will be regarded as the final classification results of ELM. For SVM, LIBSVM is employed in this paper, which is devolved by Chang and Lin [51].

KNN requires two parameters to tune: The number of neighbor k and the distance metric. In this work, the values of k vary from 1 to 20, and several distance metrics which are used are Euclidean distance, cityblock distance, cosine distance and correlation distance. The best classification accuracy of different values of k and distance metrics will be regarded as the final results of the KNN.

Table 1, Table 2, Table 3 and Table 4 list the classification results of the four feature extraction techniques and five classification models. The kernel function of KELM is set to Gaussian kernel. The bold type numbers in diagonal indicate the number of samples classified correctly, while others indicate the number of samples misclassified.

It can be observed from the above four tables that the classification accuracy of the four wounds is influenced both by different features and classification models. In general, features extracted from frequency domain can achieve better results, while features extracted from time domain do worse. It can be also seen that the classification effect of wavelet coefficients feature works best no matter what kinds of classifier are used, while peak value feature is just performs worst. QPSO-KELM always performs better than other four classifiers regardless of what kinds of features are used. SVM is invariably performs better than rest three classifiers as well. For wounds uninfected, the best performance is achieved when the wavelet feature is put into the QPSO-KELM model, where there is no sample misclassified; for wounds infected with *Staphylococcu aureus*, QPSO-KELM performs best when the peak value is used as the feature, in which there is only one sample misclassified; for wounds infected with *Escherichia coli*, the highest classification accuracy is achieved by QDA with the feature of Fourier coefficients; for wounds infected with *Pseudomonas aeruginosa*, QPSO-KELM achieves best when features are integral value and wavelet coefficients.

**Figure 4:**
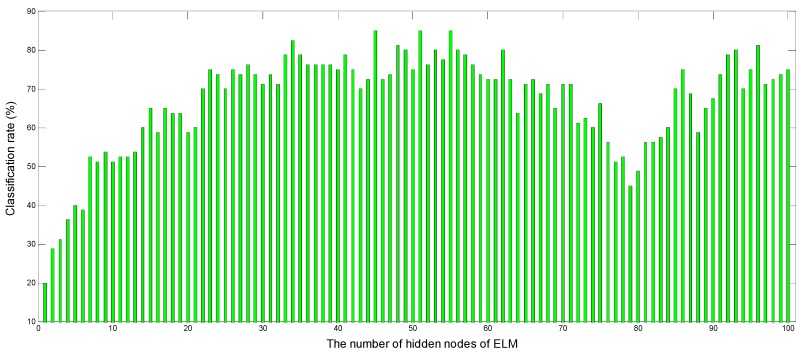
The performance of ELM according to the number of hidden nodes from 1 to 100.

**Figure 5:**
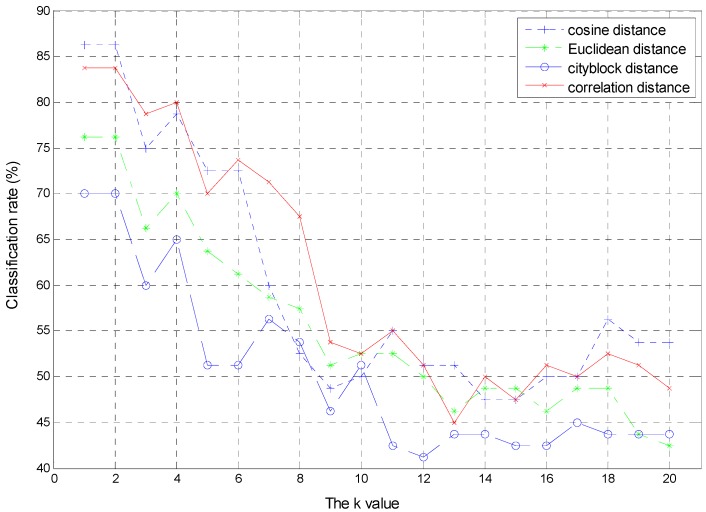
The performance of KNN with different k values and distance metrics.

Figure 4 and Figure 5 show the variation of the classification rate with the number of hidden nodes in the hidden layer of ELM and the k value of KNN for the priority to classification of wavelet coefficients feature. Figure 4 shows only the classification results of one of the 100 repeated experiments to display the change process with the number of hidden nodes in the hidden layer varying from 1 to 100. It can be clearly seen that the classification rate gradually improves with the number of hidden nodes from 1 to 34 and from 79 to 96 from the Figure 4, while the classification rate gradually declines with the number of hidden nodes from 55 to 79. Moreover, ELM can achieve the best classification accuracy of 85% when is the number of hidden nodes are 45, 51 and 55.

Figure 5 manifests that the classification rate gradually declines as the k value increases on the whole. For different distance metrics, the cityblock distance performs worst except k = 8, 10, 20. Meanwhile, the cosine distance can achieve the best classification accuracy of 86.25% at the start stage and performs best as well at the last stage.

Another three traditional optimization methods are also investigated and used to devaluate the effectiveness of the proposed model when wavelet coefficients are used as features. PSO [52], Genetic algorithm (GA) [53,54] and Grid search algorithm (GS) are employed to optimize parameters of KELM. For GA and PSO, the maximum number of iterations and the population size are also 400 and 30, respectively, which is the same as those of QPSO. For GS, the ranges of the model parameters are set according to [44].

The range of the cost parameter C and the kernel parameter of the Gaussian kernel function are both [2^–25^,2^25^], and the step length is set as 2^0.5^. Their classification performances are shown in Table 5.

It is obviously that the QPSO-KELM model obtains 95% classification rate, while other traditional methods perform worse than the proposed model from Table 5, especially that it is all predicted correctly for wounds uninfected and wounds infected with *Pseudomonas aeruginosa*. QPSO-KELM, PSO-KELM, GA-KELM and GS-KELM can only achieve 88.75%, 87.5% and 86.25% classification rates, respectively.

It is well known that the choice of kernel function plays a crucial role in recognition and generalization capability. Thus, in order to further explore the effects of different kernel functions on the QPSO-KELM model, the effects of four kinds of common kernel functions combined with wavelet features are investigated in this experiment. Their classification performances of different kernel functions are shown in Table 6.

It can be clearly concluded that the QPSO-KELM model with Gaussian kernel function performs best from Table 6, while the linear kernel function achieves the worst accuracy. Meanwhile, the performance of the polynomial kernel function is close to that of wavelet kernel function, which achieves 91.25% and 92.50% respectively. It means that the proposed model performs best in all of the above methods.

We also use the proposed model to deal with another two experimental E-nose datasets: (1) dataset of an E-nose which recognizes seven bacteria: *Pseudomonas aeruginosa*, *Escherichia coli*, *Acinetobacter baumannii*, *Staphylococcu aureus*, *Staphylococcus epidermidis*, *Klebsiella pneumoniae* and *Streptococcus pyogenes*. The classification results of various classification models based on steady-state signals of sensors are shown in Table 7. More details concerning the experiment can be found in [55]; (2) dataset of an E-nose which detects six indoor air contaminants including formaldehyde (HCHO), benzene (C_6_H_6_), toluene (C_7_H_8_), carbon monoxide (CO), ammonia (NH_3_) and nitrogen dioxide (NO_2_) and classification results are also shown in Table 8. More details include dataset generation regarding the experiment can be found in [56].

It can be clearly concluded that the proposed QPSO-KELM model achieves the best classification accuracy among all of the above classification models for different datasets. The KELM achieves the best recognition performance of 100% for the dataset in [55] and can also obtain the best recognition accuracy except the recognition rate 70% of NH_3_ for the dataset [56]. It demonstrates that the QPSO-KELM approach has outstanding generalized performance with other datasets, which efficacy does not depend on a particular dataset.

## 5. Conclusions

In this paper, a new methodology based on the QPSO-KELM model has been presented to enhance the performance of an E-nose for wound infection detection. Four kinds of features extracted from the time and frequency domains have been developed to demonstrate the effectiveness of this classification model for four different classes of wounds. It first introduces the kernel method based on extreme learning machine into the E-nose application of this paper, which provides a new idea for signal processing of E-nose data. Moreover, this paper also provides a good solution for the optimization of kernel function parameters by QPSO, which is a contraction mapping algorithm that outperforms ordinary optimization algorithms in the rate of convergence and convergence ability. Experimental tests have been carried out to verify that the proposed QPSO-KELM model can lead to a higher accuracy rate and manifest that the QPSO-KELM model can obviously enhance E-nose performance in various applications. The model in this study also provides an efficient approach in applications related to classification or prediction, not only in E-nose applications, but also in other uses.

## Figures and Tables

**Table 1:** Classification results of peak value.

Class	Predicted as *																							
	KELM				ELM				SVM				LDA				KNN				QDA			
	1	2	3	4	1	2	3	4	1	2	3	4	1	2	3	4	1	2	3	4	1	2	3	4
1	**17**	3	0	0	**16**	3	0	1	**16**	4	0	0	**14**	6	0	0	**16**	4	0	0	**15**	3	0	2
2	1	**19**	0	0	4	**14**	2	0	5	**15**	0	0	9	**11**	0	0	2	**17**	1	0	3	**16**	1	0
3	1	1	**16**	2	0	0	**14**	6	0	0	**17**	3	1	0	**16**	3	0	3	**15**	2	0	4	**13**	3
4	1	0	2	**17**	0	1	2	**17**	0	0	4	**16**	0	0	5	**15**	1	1	2	**16**	2	0	3	**15**
**Total**	86.25%				76.25%				80.00%				70.00%				80.00%				73.75%			

* 1, No-infection; 2, *Staphylococcu aureus*; 3, *Escherichia coli*; 4, *Pseudomonas aeruginosa*, similarly hereinafter.

**Table 2:** Classification results of integral value.

Class	Predicted as *																							
	KELM				ELM				SVM				LDA				KNN				QDA			
	1	2	3	4	1	2	3	4	1	2	3	4	1	2	3	4	1	2	3	4	1	2	3	4
1	**17**	3	0	0	**15**	4	1	0	**15**	5	0	0	**13**	7	0	0	**17**	3	0	0	**16**	3	1	0
2	3	**17**	0	0	6	**13**	1	0	4	**16**	0	0	8	**12**	0	0	2	**18**	0	0	2	**14**	4	0
3	1	0	**18**	1	0	1	**17**	2	0	0	**17**	3	1	0	**17**	2	0	3	**15**	2	0	0	**17**	3
4	0	0	0	**20**	0	0	3	**17**	0	0	2	**18**	0	0	4	**16**	0	1	6	**13**	0	0	4	**16**
**Total**	90.00%				77.50%				82.50%				72.50%				78.75%				78.75%			

**Table 3:** Classification results of Fourier coefficients.

Class	Predicted as *																							
	KELM				ELM				SVM				LDA				KNN				QDA			
	1	2	3	4	1	2	3	4	1	2	3	4	1	2	3	4	1	2	3	4	1	2	3	4
1	**19**	1	0	0	**17**	3	0	0	**17**	3	0	0	**16**	4	0	0	**17**	3	0	0	**18**	2	0	0
2	2	**18**	0	0	2	**18**	0	0	2	**18**	0	0	3	**17**	0	0	1	**19**	0	0	0	**15**	5	0
3	0	0	**18**	2	0	2	**16**	2	1	1	**16**	2	0	0	**16**	4	0	1	**18**	1	0	0	**19**	1
4	0	0	2	**18**	0	0	4	**16**	0	0	1	**19**	0	0	4	**16**	1	1	1	**17**	0	0	5	**15**
**Total**	91.25%				83.75%				87.50%				81.25%				88.75%				83.75%			

**Table 4:** Classification results of wavelet coefficients.

Class	Predicted as *																							
	KELM				ELM				SVM				LDA				KNN				QDA			
	1	2	3	4	1	2	3	4	1	2	3	4	1	2	3	4	1	2	3	4	1	2	3	4
1	**20**	0	0	0	**15**	4	1	0	**17**	3	0	0	**17**	3	0	0	**17**	3	0	0	**20**	0	0	0
2	2	**18**	0	0	2	**18**	0	0	3	**17**	0	0	5	**15**	0	0	3	**17**	0	0	1	**17**	2	0
3	1	0	**18**	1	0	0	**18**	2	0	0	**18**	2	1	0	**18**	1	0	0	**18**	2	0	0	**17**	3
4	0	0	0	**20**	0	1	2	**17**	0	0	1	**19**	0	0	2	**18**	0	0	3	**17**	0	0	6	**14**
**Total**	95.00%				85.00%				88.75%				85.00%				86.25%				85.00%			

**Table 5:** Comparison with different optimization methods for KELM.

Class	Predicted as *															
	QPSO				PSO				GA				GS			
	1	2	3	4	1	2	3	4	1	2	3	4	1	2	3	4
1	**20**	0	0	0	**18**	2	0	0	**18**	2	0	0	**17**	1	0	2
2	2	**18**	0	0	3	**17**	0	0	3	**17**	0	0	2	**18**	0	0
3	1	0	**18**	1	0	0	**19**	1	0	0	**19**	1	1	0	**17**	2
4	0	0	0	**20**	0	0	3	**17**	0	0	4	**16**	1	0	2	**17**
Total	95.00%				88.75%				87.50%				86.25%			

**Table 6:** Classification results of four kernel functions used in the QPSO-KELM model.

Class	Predicted as *															
	Gaussian				Linear				Polynomial				Wavelet			
	1	2	3	4	1	2	3	4	1	2	3	4	1	2	3	4
1	**20**	0	0	0	**18**	2	0	0	**18**	2	0	0	**19**	1	0	0
2	2	**18**	0	0	2	**18**	0	0	2	**18**	0	0	2	**18**	0	0
3	1	0	**18**	1	0	0	**17**	3	1	0	**18**	1	1	0	**18**	1
4	0	0	0	**20**	0	0	5	**15**	0	0	1	**19**	0	0	1	**19**
Total	95.00%				85.00%				91.25%				92.50%			

**Table 7:** Accuracy results of various feature extraction techniques and classification models for datasets in [55].

Class	Accuracy Rate (%)					
	KELM	SVM	ELM	KNN	LDA	QDA
*Pseudomonas aeruginosa*	100.00	100.00	80.00	100.00	100.00	100.00
*Escherichia coli*	100.00	100.00	100.00	100.00	100.00	90.00
*Acinetobacter baumannii*	100.00	100.00	90.00	90.00	90.00	100.00
*Staphylococcu aureus*	100.00	90.00	90.00	80.00	90.00	90.00
*Staphylococcus epidermidis*	100.00	100.00	100.00	100.00	100.00	100.00
*Klebsiella pneumoniae*	100.00	90.00	100.00	80.00	80.00	70.00
*Streptococcus pyogenes*	100.00	80.00	90.00	60.00	60.00	60.00
Average	100.00	94.29	92.86	87.14	88.57	87.14

**Table 8:** Accuracy results of various feature extraction techniques and classification models for datasets in [56].

Class	Accuracy Rate (%)					
	KELM	SVM	ELM	KNN	LDA	QDA
HCHO	94.23	94.23	92.31	90.38	94.23	63.46
C_6_H_6_	90.91	87.88	72.73	75.76	57.58	87.88
C_7_H_8_	100.00	100.00	100.00	100.00	100.00	100.00
CO	100.00	91.67	100.00	91.67	83.33	100.00
NH_3_	70.00	60.00	70.00	70.00	80.00	80.00
NO_2_	100.00	100.00	66.67	66.67	83.33	83.33
Average	92.52	88.96	83.62	82.41	83.08	85.78
